# Refractory focal seizures occurred in clusters in a girl with a *de* *novo *mutation of the *ATP6V0A1* gene: a case report

**DOI:** 10.1186/s13256-026-06069-w

**Published:** 2026-05-03

**Authors:** Longying Peng, Cuijie Wei, Yuehua Zhang, Qingping Zhang, Wenhui Wang, Xinhua Bao

**Affiliations:** 1 Department of Pediatrics, Peking University First Hospital, Beijing, China; 2 Department of Pediatrics, Affiliated Hospital of Zunyi Medical University, Zunyi, Guizhou China; 3 Department of Pediatrics, Guizhou Chidren’s Hospital, Zunyi, China; 4 Department of Neurology, Children’s Hospital of Shanxi, Taiyuan, China

**Keywords:** *ATP6V0A1* gene, Developmental delay, Focal epilepsy, Cluster seizures, Cerebral atrophy

## Abstract

**Background:**

Variants in the *ATP6V0A1* gene, which encodes the α1 subunit of the V0 domain of the V-ATPases, are associated with developmental and epileptic encephalopathy 104 (DEE104). This study aimed to characterize the clinical features of a Chinese patient with *ATP6V0A1* variants and facilitate the early diagnosis and treatment.

**Case presentation:**

We report a case of a 13-month-old Chinese girl with DEE who presented with developmental delay, acute onset of clustered focal seizures, and status epilepticus. Physical examination revealed hypotonia and microcephaly. Brain MRI showed mildly enlarged bilateral lateral ventricles, a thin corpus callosum, and progressive cerebral atrophy. Genetic analysis identified a de novo missense mutation in the *ATP6V0A1* gene (c.2222G > A, p.R741Q). While her epilepsy was refractory to multiple antiepileptic drugs, her seizures were controlled effectively with oxcarbazepine (OXC).

**Conclusions:**

We identified a patient with DEE104 carrying a de novo* ATP6V0A1* mutation, whose clinical presentation included developmental delay, clustered focal seizures, and status epilepticus. Our findings provide further support for considering OXC in the management of *ATP6V0A1*-related epilepsy.

## Background

*ATP6V0A1*, which was initially identified in 1995, encodes the a1 subunit of the V0 domain of the vacuolar ATPase (V-ATPase) that transports protons across cellular membranes to acidify various organelles [1, 2]. Recently, variants in the *ATP6V0A1* gene have been associated with developmental delay and epilepsy [3]. To date, however, only 21 patients with epilepsy associated with *ATP6V0A1* variants have been reported in 2 papers worldwide [3, 4]. Consequently, the detailed clinical manifestations and effective antiepileptic drugs for this disorder remain to be fully elucidated. Here, we report the case of a Chinese girl with DEE caused by a de novo* ATP6V0A1* variant.

## Case presentation

A 13-month-old Chinese girl, the first child of nonconsanguineous parents, was admitted. She was born at term following an uneventful pregnancy, with a birth weight of 3050 g and length of 50 cm. Her Apgar scores were 8, 10, and 10 at 1, 5, and 10 minutes, respectively. The child exhibited a mild delay in reaching gross motor milestones: head control at 3 months, rolling over at 4 months, independent sitting at 8 months, and pulling to stand (with brief independent standing) by 13 months. Language development was also delayed, with her first non-specific words (“Mama” and “Dada”) emerging around that age (13 months). Her history began at 4 months of age with recurrent clusters of focal seizures, characterized by eye deviation (left or right), bilateral eye blinking, foaming at the mouth, and facial cyanosis. These seizures occurred 2–68 times daily, each lasting approximately 10 seconds. Initially, each cluster persisted for 2–3 days before spontaneously ceasing, with recurrence every 3–5 days. Initial treatment with valproate sodium (VPA) led to partial remission. The subsequent addition of lacosamide (LCM) and nitrazepam (NZP) resulted in a brief 2-month seizure-free period. However, LCM was discontinued due to ineffectiveness, and NZP was withdrawn at 13 months due to severe hypotonia. The addition of topiramate (TPM) was ineffective. Upon admission, the patient was experiencing approximately 60 seizures per day, each lasting 3–9 minutes. This intense activity, which persisted for 5 days, was associated with a progressive decline in her level of consciousness.

**Fig. 1:**
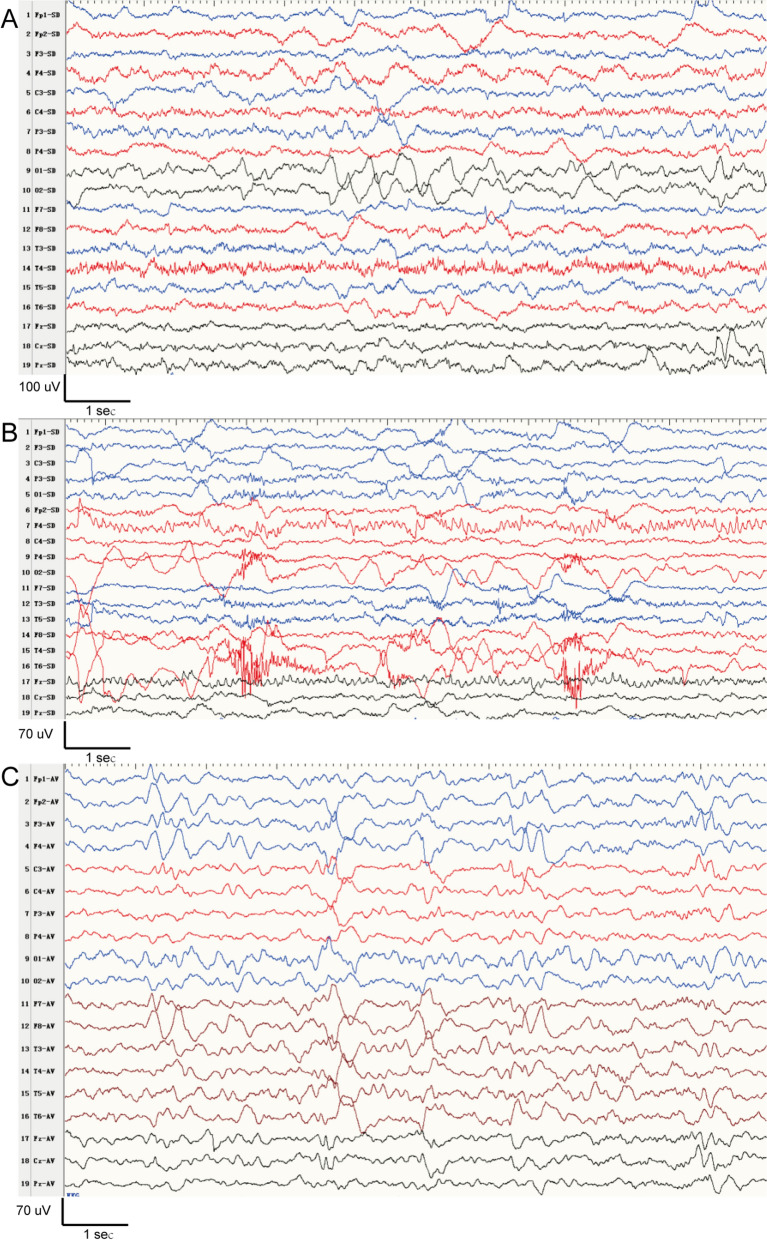
EEGs of the patient with the *ATP6V0A1* gene variant. **A**. Interictal EEG at the age of 13 months demonstrated multifocal slow wave discharges predominantly in the left posterior area. **B**. The ictal EEG showed that 10—11 Hz low-amplitude sharp waves arose from F4 and Fz. **C**. EEG at 16 months of age demonstrated multifocal epileptiform discharges

**Fig. 2:**
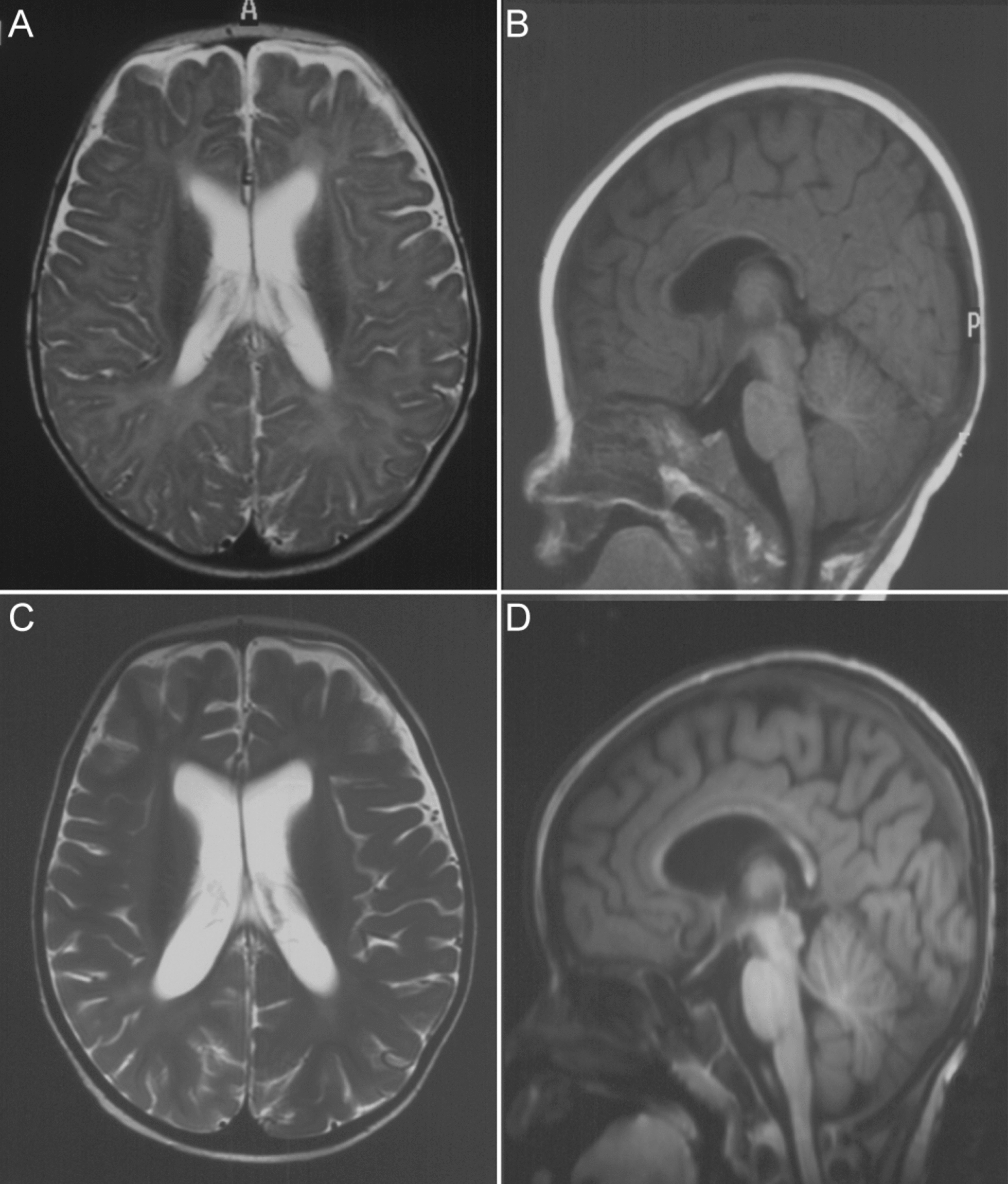
Cranial MR image of the patient with the *ATP6V0A1* variant. MRI performed at 4 months of age showed mild enlargement of the bilateral ventricles with a thin corpus callosum (**A**, **B**). MRI performed at 13 months of age showed progressive cerebral atrophy (**C**, **D**). A and C are T2-weighted images, and B and D are T1-weighted images

On admission, physical examination revealed microcephaly (head circumference 43 cm, < 2nd percentile) and mild hypotonia. Neurological examination showed that the hypotonia was axial and appendicular, resulting in poor head control while sitting. Deep tendon reflexes were normal. Cranial nerve examination, including pupillary light reflexes, was unremarkable. No dysmorphic facies were noted. Developmental assessment using the Gesell Developmental Schedule indicated mild global developmental delay. Blood and urine metabolic screening and routine biochemical tests were unremarkable. Electroencephalogram (EEG) revealed multifocal 3–5 Hz slow wave discharges predominantly in the left posterior area (Fig. 1A). The ictal EEG showed that 10–11 Hz low-amplitude sharp waves arose from F4 and Fz, lasting for approximately 50 seconds (Fig. 1B). Cranial magnetic resonance imaging (MRI) at 5 months of age revealed mild enlargement of the bilateral ventricles with a thin corpus callosum. By 13 months of age, progressive cerebral atrophy was noted (Fig. 2).

**Fig. 3:**
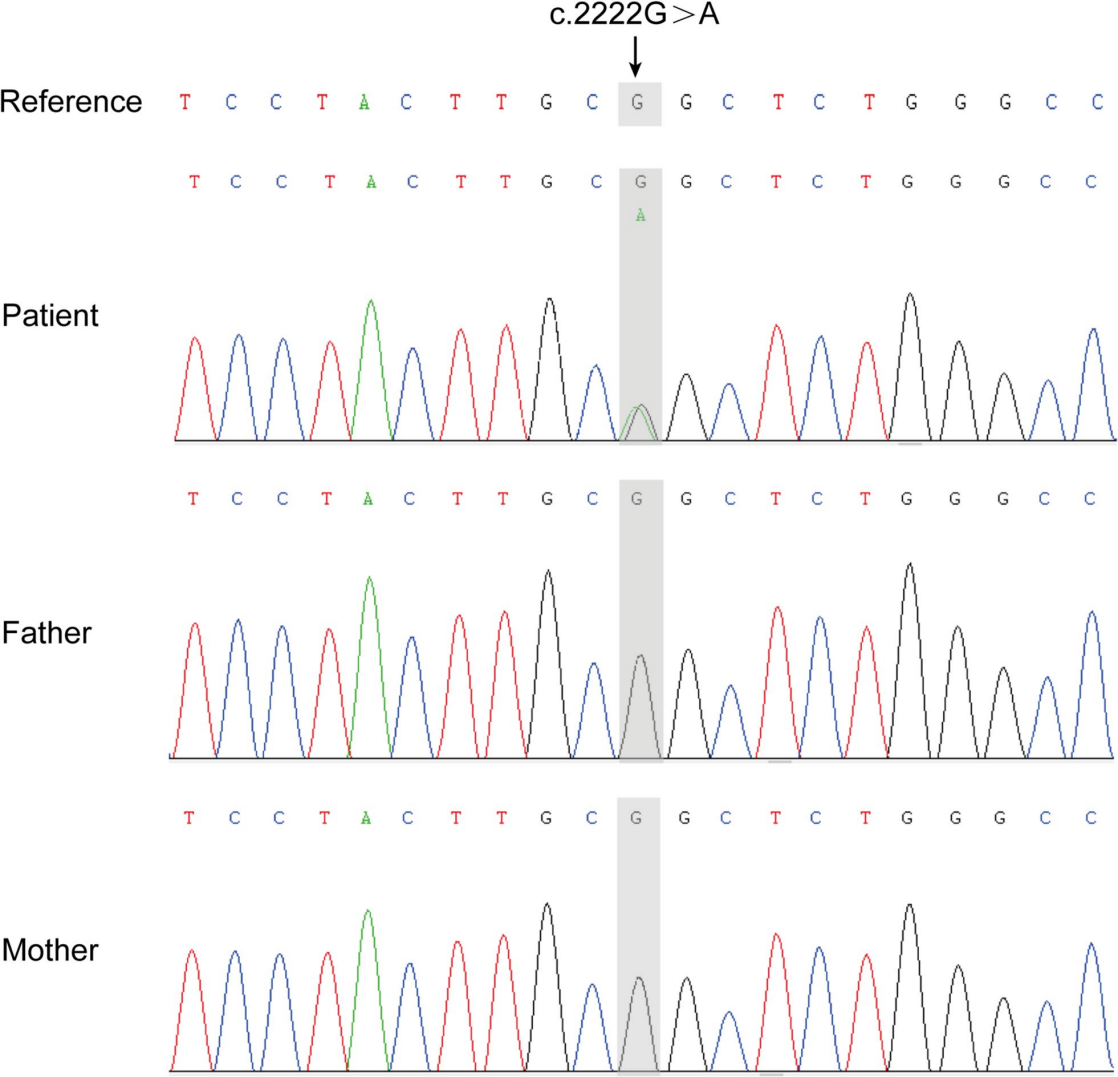
Mutation in the *ATP6V0A1* gene: A missense variant (NM-001130020.1: c.2222G > A) was detected in the patient, while a wild type was detected in the parents

Whole-exome sequencing (WES) was performed for the patient and her parents. A de novo* ATP6V0A1* missense variant (NM_**_**_001130020.1: c.2222G > A, p.R741Q) was identified in the patient, which was confirmed by Sanger sequencing (Fig. 3). This variant was classified as pathogenic according to the ACMG guidelines [5], based on the following criteria: PS2 (confirmed de novo status), PM2 (absent in population databases), and PP3 (supporting evidence from multiple computational predictors for a deleterious effect). To our knowledge, *ATP6V0A1*-related epilepsy has not been previously reported in the Chinese population, based on searches of public databases including the China National Knowledge Infrastructure (CNKI), Wanfang database, and Chinese Biomedical Database (CBM).

After admission, the patient experienced frequent focal seizures and remained unconscious during the interictal periods. Intravenous midazolam was administered, but failed to achieve complete seizure control. Subsequently, oxcarbazepine (OXC) was initiated at a dose of 7.5 mg/kg/day. Within three days of treatment at this dose, the seizure frequency markedly decreased. After the OXC dose was titrated up to 22 mg/kg/day, the patient became seizure-free. At the most recent follow-up (age 16 months), she remains seizure-free. She is able to sit independently and walk with assistance. The interictal EEG showed multifocal epileptiform discharges (Fig. 1C).

## Discussion

The *ATP6V0A1* gene, located on 17q21.2, encodes the brain-enriched α1 isoform of the membrane-bound V0 component of the V-ATPase complex [2]. The V-ATPase plays a pivotal role in acidification and protein degradation in lysosomes, as well as in the maintenance of neuronal homeostasis [3, 6, 7]. Dysfunction of the V-ATPase affects lysosomal acidification, which leads to reduced synaptic connectivity, decreased neurotransmitter content in synaptic vesicles, impaired activity of various lysosomal enzymes, and disrupted substrate clearance. These deficits may collectively contribute to a range of neurological disorders [6–8]. In this context, it is important to note that *ATP6V0A1* is not an isolated epilepsy gene but part of a larger family of V-ATPase subunit genes (*ATP6V1A, ATP6V1B2, ATP6AP2*) in which mutations cause overlapping phenotypes of developmental delay, intellectual disability, and epilepsy. These disorders, sometimes collectively referred to as “V-ATPaseopathies,” likely share a core pathophysiology [9]. Therefore, *ATP6V0A1*-related DEE 104 should be clinically and mechanistically considered within this broader diagnostic and pathogenic framework.

Variants in the *ATP6V0A1* gene have been recently associated with neurological disorders [3]. Two major inheritance patterns have been observed: inherited biallelic variants associated with neurodevelopmental disorders including epilepsy and brain atrophy, and de novo missense variants causing developmental and epileptic encephalopathy 104 (DEE104). The majority of DEE104 cases are caused by de novo mutations [3]. Our patient carries a de novo heterozygous mutation, p.R741Q. This variant constitutes a mutational hotspot, accounting for over 70% of de novo cases in DEE104 [3, 4]. To our knowledge, *ATP6V0A1*-related epilepsy has not been previously reported in the Chinese pediatric population. Here, we report the first Chinese case, a girl with the hotspot p.R741Q variant, expanding the ethnic spectrum of this disorder.

To the best of our knowledge, only 21 patients with epilepsy due to *ATP6V0A1* variants have been reported in the literature across 2 studies, with 19 of them experiencing seizure onset within the first year of life. All reported patients share common core features, including epilepsy and varying degrees of developmental delay [3, 4]. Some patients also present with additional findings such as ataxia, microcephaly, hypotonia, enlarged lateral ventricles, and progression of cerebral atrophy [4]. The clinical phenotype of our patient is consistent with this spectrum.

Refractory seizures have been reported in patients with the de novo p.R741Q variant in the *ATP6V0A1* gene [3]. The seizure types included focal and generalized tonic‒clonic seizures. However, detailed manifestation and treatment response were not well documented. Here, we describe in detail the seizure characteristics and response to anti-seizure medications in a patient with this mutation. She had brief focal seizures occurring in clusters since 4 months of age. By the time of admission, these clusters had progressed to potentially life-threatening status epilepticus. To the best of our knowledge, this progression from clustered seizures to status epilepticus has not been previously reported in ATP6V0A1-related disorders. Our findings expand the understanding of the neurological phenotype associated with this variant.

Regarding antiseizure medication, our patient was refractory to multiple agents, including VPA, NZP, and TPM, but showed a marked and sustained response to OXC. To our knowledge, similar responses to OXC have not been reported in other *ATP6V0A1*-related cases, so this represents an isolated observation. The therapeutic effect of OXC in ATP6V0A1-related epilepsy is likely mediated by its downstream modulation of neuronal hyperexcitability, rather than correcting the primary V-ATPase dysfunction. Pathogenic ATP6V0A1 variants impair synaptic vesicle acidification, synaptic vesicle cycling, and neurotransmitter release, leading to network instability and a predisposition to synchronized discharges [4, 9]. As a voltage-gated sodium channel blocker, OXC suppresses this secondary hyperexcitability by inhibiting sustained neuronal firing and preventing the spread of abnormal activity, thereby providing symptomatic seizure control despite the underlying genetic defect. This single-case observation suggests that OXC may be an effective therapeutic option for *ATP6V0A1*-related epilepsy, particularly in patients harboring the p.R741Q variant. Therefore, OXC should be considered in the treatment regimen for such patients. Further studies are needed to validate its efficacy in a larger cohort.

## Conclusions

Here, we present the detailed clinical course of a Chinese girl with a de novo p.R741Q mutation in the *ATP6V0A1* gene. The patient manifested global developmental delay, recurrent seizure clusters, and status epilepticus. Her epilepsy was refractory to multiple anti-seizure medications but showed a marked and sustained response to OXC. Our findings suggest that seizure clusters progressing to status epilepticus may represent a severe phenotype associated with this variant. Furthermore, the positive response to OXC in this case highlights its potential as a therapeutic option for *ATP6V0A1*-related epilepsy, warranting further investigation.

## Data Availability

The original contributions generated for the study are included in the articles, and further inquiries can be directed to the corresponding author.

## Abbreviations

CBM: Chinese Biomedical Database
CNKI: China National Knowledge Infrastructure
DEE: Epileptic encephalopathy
EEG: Electroencephalogram
LCM: Lacosamide
MRI: Magnetic resonance imaging
NZP: Nitrazepam
OXC: Oxcarbazepine
TPM: Topiramate
V-ATPase: Vacuolar ATPase
VPA: Valproate sodium
WES: Whole-exome sequencing
